# Neural Correlates and Sex‐Specific Effects of Affectively Driven Processes Underlying Decision‐Making in Adult ADHD

**DOI:** 10.1002/brb3.70215

**Published:** 2025-02-28

**Authors:** Eva Halbe, Alec Jamieson, Moritz Bergmann, Aylin Mehren, Ben J. Harrison, Christopher G. Davey, Tony Stöcker, Alexandra Philipsen, Silke Lux

**Affiliations:** ^1^ Department of Psychiatry and Psychotherapy University of Bonn Bonn Germany; ^2^ Department of Psychiatry The University of Melbourne Melbourne Australia; ^3^ Department of Cognitive Neuroscience, Donders Institute for Brain, Cognition and Behavior Radboud University Nijmegen Medical Centre Nijmegen The Netherlands; ^4^ German Center for Neurodegenerative Diseases (DZNE) Bonn Germany

**Keywords:** attention‐deficit/hyperactivity disorder, decision‐making, functional magnetic resonance imaging, sex/gender differences

## Abstract

**Aims:**

Adults with attention‐deficit/hyperactivity disorder (ADHD) often exhibit heightened risk‐taking behavior due to disadvantageous decision‐making. This study investigates the influence of preceding unconscious and affectively driven processes on this behavior, with a focus on sex‐specific effects.

**Methods:**

Functional magnetic resonance imaging was used to examine neural activity during the anticipation phase of decision‐making in 18 individuals with ADHD (10 females and 8 males) and 20 healthy controls (10 females and 10 males) using a modified version of the Balloon Analogue Risk Task.

**Results:**

During the anticipation of decision‐making, individuals with ADHD exhibited reduced activation in the right precuneus and the right superior frontal gyrus compared to healthy controls. Sex‐specific effects were exclusively observed within the ADHD group, showing increased neural activity in females compared to males in areas including the dorsolateral prefrontal cortex, left insula, right caudate, right cuneus, and precuneus.

**Conclusion:**

These findings indicate altered neural activity in adult patients with ADHD with sex‐specific differences during the anticipation of a risky decision. The study underscores the importance of the right precuneus and superior frontal gyrus in relation to metacognitive functioning and interoceptive awareness. However, further research is needed to explore the interplay of unconscious processes during decision‐making in ADHD.

## Graphical abstract

Neural differences in the right precuneus and right superior frontal gyrus in patients with ADHD are present during the anticipation of decision‐making. Gender‐specific differences also indicate variability in decision‐making behavior within the patient group. These findings shed light on metacognition and interoceptive perception and emphasize the complexity of ADHD.

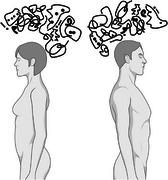

## 1 Introduction

Attention‐deficit/hyperactivity disorder (ADHD) is a neurodevelopmental disorder characterized by behavioral and cognitive impairments across attention, concentration, impulsivity, and motor domains (American Psychiatric Association [APA] 2013; Faraone et al. 2000; Polanczyk et al. 2014). While diagnosis typically occurs during childhood, these impairments often persist into later life, resulting in a prevalence of around 4.4% in adult samples (Faraone, Biederman, and Mick 2006; Gerhand and Saville 2022; Kessler et al. 2006; Polanczyk et al. 2014; Waltereit, Ehrlich, and Roessner 2023). This, in turn, can have lifetime negative impacts on mental health and socioemotional functioning (Beheshti, Chavanon, and Christiansen 2020). A common behavioral characteristic of adult patients with ADHD is heightened risk engagement; these occur in several life domains, including gambling, substance use, driving, and sexual behavior (Faregh and Derevensky 2011; Flory et al. 2006; Rooney, Chronis‐Tuscano, and Yoon 2012; Thompson et al. 2007). In this context, the observed risk‐taking can often be traced back to disadvantageous decision‐making (DM) behavior (Dekkers et al. 2016; Pollak et al. 2019). To better characterize the cognitive constructs underlying this behavior, research has predominantly focused on feedback perception, impulsivity, and disinhibition due to being core features of ADHD (Scheres et al. 2007; Ströhle et al. 2008; Winstanley, Eagle, and Robbins 2006). However, it is rarely considered that risk engagement is frequently a result of quick and intuitive decisions primarily driven by affective processes (Mäntylä et al. 2012; Sonuga‐Barke 2002), which are also known to be altered in ADHD (Beheshti, Chavanon, and Christiansen 2020; Kutscheidt et al. 2019; Materna et al. 2019; Shushakova, Ohrmann, and Pedersen 2018; Wender, Wolf, and Wasserstein 2001).

The relationship between affective functions and disadvantageous DM behavior was first described by the somatic marker hypothesis (Damasio 1996). In this context, a perceived stimulus is unconsciously associated with an individual emotional value, triggering a change in autonomic nervous system activity. This change elicits a physiological response that can be quantified, for instance, by an increased heart rate, altered respiration, or sweating (Figner and Murphy 2011). Consequently, the interconnection of stimulus, emotional value, and physiological activity can be stored as a somatic marker and guides unconscious DM behavior. Thus, re‐confrontation with a familiar stimulus elicits a quick bodily response, which is often perceived as a sense of intuition and may unconsciously control behavior (Christopoulos, Uy, and Yap 2019; Damasio 1996). While risk‐taking is frequently associated with externalizing behaviors, internalizing processes also appear to contribute to this behavior through affective functioning. These contrasting characteristics could be used to differentiate the symptoms of ADHD between male and female patients. Study results indicate that male patients with ADHD are more likely to show symptoms reflecting externalizing behaviors, whereas female patients are more likely to be affected by internalizing difficulties (Gaub and Carlson 1997; Gershon 2002). As a result, boys are often diagnosed at earlier developmental stages than their female peers' age due to their conspicuous and more stereotypical behavior (Klefsjö et al. 2021). This could not only affect the later course of the disorder in “overlooked” girls but also lead to a distorted clinical picture of ADHD and a sex publication bias in research. Consequently, findings are often generalized across both sexes in clinical contexts, with research focusing more on externalizing behaviors than on emotionally driven functioning in ADHD. Study results also indicate that females have a greater tendency to develop compensatory behaviors and coping strategies, which might mask ADHD‐related symptoms (Mowlem et al. 2019). Taken together, research on sex‐related differences in the symptomatology of ADHD is limited and potentially influenced by bias in the existing literature. Therefore, it remains unclear to what extent sex impacts affectively driven DM behavior in ADHD.

At a neural level, the interconnection of somatic markers was first observed in patients with lesions in the area of the ventromedial prefrontal cortex (vmPFC). Following vmPFC damage, these patients were observed to have deficits in DM behavior concerning future advantageous outcomes (Bechara et al. 1994). Studies investigating the neural components of somatic marker processing have identified several key regions that are hypothesized to contribute to various cognitive functions, including memory, emotion, and executive functioning. Neuroimaging studies have found activation patterns in the dorsolateral prefrontal cortex (DLPFC), amygdala, anterior insula, and posterior cingulate cortex (PCC) that are associated with the anticipatory, unconscious generation of a decision (Li et al. 2010, 2020; Rao et al. 2008; Tannou et al. 2021; Wang et al. 2022). It is suggested that to achieve this, the emotional evaluation of a present stimulus is generated by a quick representation of previous similar experiences. In this context, memory and emotion are integrated and trigger a change in the somatic state. Studies on lesion patients have additionally suggested that disadvantageous DM behavior is dependent on the inadequate integration by the vmPFC (Li et al. 2010). Further studies investigating the integrative role of the frontal lobe have found that the orbitofrontal cortex (OFC) may also be a central component in advantageous DM. Both areas were found to receive afferent input from the designated neural systems and to be responsible for eliciting a visceral response (Bechara et al. 2005; Verdejo‐García and Bechara 2009). In the form of a feedback loop, this bodily response is subsequently perceived by neurons located in the brainstem. By activation of dopaminergic neurons all over the cortex and striatum, synaptic activity was found to modulate the initiation of a behavioral action (Hervé et al. 1987; Li et al. 2010; Verdejo‐García and Bechara 2009).

In research on behavioral problems in ADHD, the impact of unconscious processes that proceed with DM was rarely considered, and previous studies have predominantly focused on neural mechanisms of feedback processing. In this context, study results indicate that deviations in frontostriatal circuits and prefrontal cortex (PFC) functioning are associated with lower dopamine levels and are linked to behavioral deficits in ADHD (Seidman, Valera, and Makris 2005). In this context, reduced functional connection between the PFC and the striatum was identified as the cause of disturbances in the evaluation of choices and the regulation of behavior in ADHD (Cubillo et al. 2012; Dickstein et al. 2006; Tamm et al. 2004). Furthermore, it has been shown that the stimulation of the left DLPFC and the right vmPFC improves clinical symptoms of emotional processing and reduces risky DM in children with ADHD (Nejati et al. 2020). Overall, most findings underscore the significant role of the frontoventral striatal reward circuits and the mesolimbic dopamine pathways, which are crucial for reward processing, motivation, and the anticipation of future outcomes (Averbeck and O'Doherty 2022; Cox and Witten 2019). In addition, the impact of autonomic nervous system activity has also been studied in patients with ADHD and was identified as impaired. Across multiple physiological measures, including heart rate, skin conductance, and pupillometry, results indicate that ADHD is associated with hypoarousal of the autonomic nervous system both at rest and during emotional tasks (Bellato et al. 2020). Moreover, in a previous study, we found that alterations in autonomic affective responses are related to subsequent abnormalities in DM behavior in adult patients with ADHD (Halbe et al. 2023). Further investigations showed that sex has a strong effect on differences in physiological activity and behavior between ADHD and healthy controls (HC), with females being particularly impaired in affectively driven DM behavior (Halbe et al. 2024).

Taken together, the body of literature suggests that functionality in somatic‐emotional processes that precede a decision and guide unconscious behavior are impaired in ADHD and, furthermore, that these abnormalities may be sex‐dependent. However, so far, there has been no study examining the neural preceding process of DM in adult patients with ADHD. This study builds on the findings of our previous research study that indicated the impact of affective processing during the performance on a modified version of the Balloon Risk Task (BART) (Halbe et al. 2023). This version of the BART was designed to address intuitive DM that demands a high emotional–motivational effort during the performance. In comparison to the original version of the BART, this version was conceptualized to decrease cognitive effort and to increase intuitive affectively driven behavior, as risk‐taking is operationalized as the latency time to stop the inflation of an automatically growing balloon with a restricted time window for DM. This means that a decision must be made between continuing‐to‐inflate and actively stopping‐further‐inflation. Thus, longer latency times are associated with increased risky DM behavior (Halbe et al. 2023; Henn et al. 2023). To extend our previous findings and understanding of emotional–motivational behavior in ADHD, the aim of the current study is to further explore neural correlates during the anticipation of a risky DM, measured by functional magnetic resonance imaging (fMRI). We used the modified BART to compare neural activity patterns between patients with ADHD and HC before a decision was made. Analyses of changes in blood oxygenation level‐dependent (BOLD) signals in anticipation of a decision were used to reveal insights into the integrative processes of somatic marker functioning. Furthermore, sex was considered exploratively to allow a more extensive exploration of underlying processes of affective functions related to risky DM behavior in adult ADHD.

## 2 Materials and Methods

### 2.1 Participants

A total of 21 adult patients with ADHD and 21 HC were recruited from the outpatient clinic of the Department of Psychiatry of the University Hospital Bonn and by public advertisement via flyers and social media. Patients met DSM‐V criteria for adult ADHD (APA 2013) and were quantified in the severity of their symptoms based on the Conners Adult Rating Scale (Conners et al. 1999) and the validated short version of the Wender Utah Rating Scale (Retz‐Junginger et al. 2002). All participants underwent a brief diagnostic interview (Mini‐DIPS; Margraf and Schneider 2009) to assess their mental health status and comorbidities and a survey regarding demographic information. In the patient group, medication intake was assessed through this survey and they were asked to discontinue use for 24 h before their participation in the study. Participants were excluded if they met any of the following criteria: neurological diseases, affective or psychotic disorders, participation in our preliminary study, under 18 or over 60 years of age. All participants underwent a medical preexamination regarding physical and mental suitability for MRI measurement. Classification regarding sex differences was based on biological sex. The study was approved by the Ethics Committee of the Medical Faculty of the University of Bonn (122/21) and all participants gave oral and written informed consent. Each subject was assigned a subject study ID. Further processing and analysis of the data was carried out exclusively using the assigned ID to minimize subjective bias on the part of the experimenter.

### 2.2 Behavioral Task

The paradigm used in the current study is a modified version of the Balloon Analogue Risk Task (BART; Lejuez et al. 2002) that was conceptualized to demand affective‐motivational–driven behavior (Halbe et al. 2023; Henn et al. 2023). The paradigm was performed using Presentation software (Version 23.0, Neurobehavioral Systems Inc., Berkley, CA, www.neurobs.com). The modified design of the BART requires more intuitive DM, which has been shown to be closely linked to emotional–motivational processing due to the automatically inflating balloon (no active pumping) (Henn et al. 2023). The modified BART includes 60 trials divided into low‐rewarded (*n* = 30) and high‐rewarded (*n* = 30) trials. The reward condition is visibly displayed at the beginning of each trial. Participants were instructed that the increasing size of the automatically inflating balloon on the screen not only coincides with an increasing amount of virtual money but also with the probability of explosion of the balloon. An explosion of the balloon causes a loss of the already collected money. Thus, participants are asked to specify a time point at which the money that has been collected so far should be saved by pressing a response button. Regardless of the outcome, the explosion of the balloon was not visibly shown to the participants. The dynamic inflation of the balloon is presented on the screen in each trial for 5 s, followed by a fixation cross (250 ms) and the feedback display (2500 ms). In case of successful saving of the money, by pressing the response button before the explosion of the balloon, the amount of money gained is displayed. In case the balloon has already burst at the determined time‐point, negative feedback appears, informing that no money could be saved. Following this, another fixation cross (250 ms) and the next reward condition were presented. For further information on the BART (see Henn et al. 2023). Disadvantageous DM toward risk engagement is assessed by response time (RT); longer RTs indicate higher levels of risk‐taking behavior.

### 2.3 Data Acquisition

The MRI measurements were conducted using a Siemens 3T scanner (Magnetom Skyra, Siemens, Erlangen, Germany) equipped with a 32‐channel head array for whole‐brain high‐resolution signal reception. All MRI measurements were performed at the Institute for German Center for Neurodegenerative Diseases (DZNE) in Bonn. Functional images, which captured the BOLD signal, were acquired using a rapid three‐dimensional echo planar imaging sequence that combined blipped‐CAIPI acceleration and water‐selective excitation for fat suppression (Stirnberg et al. 2017) with the following parameters: repetition time 1500 ms, echo time 30 ms, flip angle 16°, matrix size 120 × 120 × 78, voxel size 1.8 × 1.8 × 1.8 mm^3^, oblique‐axial slice orientation approximately along the anterior–posterior commissure, anterior–posterior phase encoding direction, slice parallel imaging acceleration factor 6, CAIPI shift 2. The imaging parameters were selected as a feasible compromise between sufficiently high whole‐brain sampling rate with moderate parallel imaging noise penalty, on the one hand, and reduced intra‐voxel dephasing (signal drop‐outs) compared to typically larger voxels in particularly sensitive areas such as the vmPFC and OFC, on the other hand. Each participant completed 60 trials of the modified BART, resulting in a total duration of 12 min. After the functional scans, individual high‐resolution T1‐weighted anatomical images were obtained using a multi‐echo magnetization‐prepared rapid gradient echo (ME‐MP‐RAGE) sequence that combined CAIPIRINHA acceleration and elliptical sampling (Brenner et al. 2014) with the following parameters: repetition time 2560 ms, inversion time 1100 ms, flip angle 7°, matrix size 320 × 320 × 224, voxel size 0.8 × 0.8 × 0.8 mm^3^, sagittal slice orientation, slice parallel imaging acceleration factor 2, CAIPI shift 1, Turbofactor 180, acquisition time 6 min 52 s. The root‐mean‐squares combination of the four echo time images (1.85, 3.75, 5.65, 7.55 ms) was subsequently used as the anatomical image. Using the software presentation, the paradigm was projected onto a display screen at the back of the scanner, and participants viewed the task through a mirror that was fixated on top of the coil. Responses within the task were made by pressing a button on a joystick using the thumb of the right hand.

### 2.4 Data Analysis

Preprocessing and analyses of the functional MRI data were conducted using Statistical Parametric Mapping software v7219 (SPM12, Functional Imaging Laboratory, London, UK). The functional images were realigned to make corrections for head movements and were corrected for slice acquisition time differences. Motion parameters for each participant were estimated using Motion Fingerprint (Wilke 2012). Participants with motion values exceeding 3 mm in terms of total displacement (TD) or mean slice‐to‐slice (STS) were excluded from further analyses. Furthermore, to ensure that motion‐induced artifacts do not affect group differences in neural activation patterns, we tested whether TD motion parameters differed between groups. The corrected images were coregistered with the anatomical image and normalized to the standard Montreal Neurological Institute (MNI) brain template. Smoothing was applied with a Gaussian kernel (5‐mm full width at half‐maximum) to improve statistical power. Note that this does not undermine the reduced intra‐voxel dephasing that has already been established by relatively small voxels during image acquisition. To remove low‐frequency fluctuations, a high‐pass filter with a cut‐off at 128 Hz was applied.

For the fMRI data analysis, an event‐related design was utilized. Therefore, all event triggers of the BART were included as regressors in first‐level analyses capturing the timing of the appearance of the balloon and the display of the feedback in relation to the reward condition (high; low) and the outcome (pos; neg) in the previous trial: high_pos_start; low_pos_start; high_neg_start; low_neg_start; pos_feedback; neg_feedback. All further analysis steps were performed with respect to the anticipatory phase of a DM. Regarding the research question of the current study, all anticipatory regressors were considered as one to determine the contrast image “Start.” In addition, motion parameters from the preprocessing were incorporated as multiple regressors of no interest in the analyses. All contrast images were used to perform a two‐sample *t*‐test (ADHD vs HC) for group‐level analyses. An initial whole‐brain voxel threshold of 0.001 uncorrected with no restriction to cluster size was applied. Significances in neural activity changes were considered for multiple testing on cluster level (corrected *p*_FWE_ < 0.05). Peak beta values were calculated for significant activation patterns between groups, which were subsequently analyzed for a correlative relationship with ADHD symptoms using Spearman correlation analysis. Furthermore, a Spearman correlation was used to analyze the correlation between motion parameters and beta values.

An additional 2 × 2 full factorial analysis was performed, including group (ADHD and HC) and sex (female and male) as factors to explore sex‐dependent effects during the anticipation of a DM. Brain regions were labeled using the toolbox Automated Anatomical Labelling 3 (AAL3; Rolls et al. 2020) in Matlab R2022a (The MathWorks Inc.). Behavioral data regarding the performance in the BART were analyzed using SPSS Statistics 22 (IBM, Armonk, NY, USA). A two‐way analysis of variance (ANOVA) was conducted to examine the effects of sex and group on the mean of RT based on the data from 60 trials per participant. Sex (male and female) and group (ADHD and HC) were considered as independent variables, while RT served as the dependent variable. Assumptions of normal distribution and homogeneity of variances were assessed before conducting the ANOVA. RT of both reward conditions (low and high) were considered together so that the behavioral data were considered independent of the reward level.

## 3 Results

### 3.1 Risky DM Behavior

In total, 38 participants (*n* = 20 HC, *n* = 18 ADHD) were included in the following analyses of behavioral and fMRI data. One HC and two patients with ADHD were excluded due to excessive movement parameters. Another patient with ADHD was excluded due to severe comorbid affective symptoms. There were no significant differences between the groups in age, sex, and educational level. The average age in the ADHD group was 30.83 (ranging from 19 to 57) and 28.45 (ranging from 22 to 48) in the HC group. Patients with ADHD scored significantly higher on the clinical questionnaires in terms of ADHD symptomatology and depression (see Table 1). Sixteen patients with ADHD took stimulant medications (Elvanse, Medikinet, methylphenidate, and Ritalin) regularly for a period of at least 2 weeks. These medications were discontinued 24 h before participation in the study. All participants completed 60 trials of the modified version of the BART. To investigate risky DM behavior, RT during the DM phase of the paradigm was compared between groups and sex. On average, females in the HC group showed an RT of 1610.74 ms (SD = 497.64 ms), whereas males in the HC group showed an RT of 1970.19 ms (SD = 447.61 ms). In the group of patients with ADHD, mean RT was 1538.09 ms (SD = 441.45 ms) for females and 2148.07 ms (SD = 748.55) for males. Using a two‐way ANOVA, no significant effect of group was identified (*F*_(1,34)_ = 0.091, *p* = 0.76, *η* ^2^ = 0.003), whereas a significant effect of sex was found (*F*_(1,34)_ = 7.74, *p* = 0.009, *η* ^2^ = 0.186). With no significant effect of the interaction of group and sex (*F*_(1,34)_ = 0.52, *p* = 0.477, *η* ^2^ = 0.015), sex differences in RT did not appear to differ between groups.

**TABLE 1:** Demographic, clinical, and behavioral variables.

Parameter		Median (IQR)		Mann–Whitney *U*‐test	
		HC (*n* = 20)	ADHD (*n* = 18)	*U*	*p*
Age (years)		26 (6)	27.5 (14)	175	0.883
Education (years)		18 (2)	17.5 (7)	116.5	0.488
CAARS	Hyperactivity	10 (5)	20 (14)	10.5	< 0.001
	Inattention/memory	6 (10)	23 (7)	11.5	< 0.001
	Impulsivity	7 (6)	19 (11)	30.5	< 0.001
	Self‐conception	6 (7)	12 (9)	73.5	0.003
	Total score	6 (9)	32 (16)	7.0	< 0.001
WURS‐k		10 (18)	31 (10.5)	25	< 0.001
BDI		4 (5)	7 (6)	94	0.018
		Frequency		*χ* ^2^ test	
Sex (m/f)		10/10	8/10		*χ* ^2^ = 0.117

*Note*: The median was used as a measure of central tendency due to the non‐normally distributed nature of the variables. Interquartile ranges (IQR) are reported in brackets.

Abbreviations: BDI, Beck Depression Inventory; CAARS, Conners Adult ADHD Rating Scale; WURS‐k, Wender Utah Rating Scale

### 3.2 Functional MRI Data

Analysis of group differences related to TD motion parameters did not reveal a significant difference between groups (*F*_(1,36)_ = 0.109, *p* = 0.743), providing evidence that motion‐related effects do not impact group‐level results. The main focus of the analyses was to examine brain activation patterns during the anticipation phase of DM using the contrast “Start.” For the HC group, the whole brain analysis revealed significant activation in the frontal lobe, including the right inferior and bilateral middle frontal gyri as well as bilateral middle parts of the temporal lobe. Further significant activation patterns were found in bilateral postcentral, precentral, and left paracentral gyri. Subcortical activation patterns were observed in the left putamen and right caudate (see Table 2). Moreover, the cluster with peak activation in the left precentral gyrus was found to extend into the region of the left insula (see Figure S1; Table 1). For the ADHD group, the whole brain analysis revealed activation in the right middle part of the cingulate, left middle frontal gyrus, and right middle temporal gyrus (see Table 2). Activation in the cingulate also extended into the right insula (see Figure S1; Table 1).

**TABLE 2:** Brain activations associated with the anticipation of decision‐making within groups (HC and ADHD) and compared between groups (HC > ADHD; ADHD > HC).

	Peak MNI coordinates			*p*_FWE‐corr_ (cluster level)	*Z*_E_ (peak‐level)	Cluster size
	*x*	*y*	*z*			
HC						
R. Inferior frontal gyrus, opercular part	60	12	16	< 0.001	5.75	335
L. Middle frontal gyrus	4	10	50	< 0.001	5.73	8343
L. Precentral gyrus	−36	14	8	< 0.001	5.3	1039
L. Putamen	−24	6	−6	< 0.001	5.05	496
R. Middle temporal gyrus	46	−60	6	< 0.001	5.05	290
R. Postcentral gyrus	56	−12	24	< 0.001	4.97	250
L. Middle temporal gyrus	−50	−60	10	< 0.001	4.87	250
R. Caudate	15	8	−2	< 0.001	4.72	563
R. Middle frontal gyrus	36	38	30	< 0.001	4.64	757
L. Paracentral lobule	−6	−30	60	< 0.001	4.62	544
R. Crus II of cerebellar hemisphere	44	−78	−36	< 0.001	4.54	754
L. Postcentral gyrus	−66	−14	10	0.002	4.52	209
L. Crus I of cerebellar hemisphere	−52	−64	−34	< 0.001	4.49	319
L. Lobule VIII of cerebellar hemisphere	−24	−64	−54	< 0.001	4.14	275
R. Postcentral gyrus	30	−42	52	0.031	4.07	123
R. Precentral gyrus	38	−14	60	0.026	4.06	128
ADHD						
R. Middle cingulate and paracingulate gyri	22	6	20	0.001	5.3	241
L. Middle frontal gyrus	−34	40	18	0.001	4.7	231
R. Middle temporal gyrus	46	−66	2	0.021	4.63	133
HC > ADHD						
R. Precuneus	16	−40	56	0.01	4.36	154
R. Superior frontal gyrus, dorsolateral	20	60	6	0.041	4.23	115
ADHD > HC						
	Not significant					

*Note*: Initial voxel threshold was set as uncorrected (*p* < 0.001) for within‐group and between‐group comparisons. Reported activation patterns are FWE‐corrected (*p* < 0.05) on cluster level. See Supporting Information for further details of significant clusters. Cluster size reported as number of voxels.

Abbreviations: L, left hemisphere; R, right hemisphere.

**FIGURE 1:**
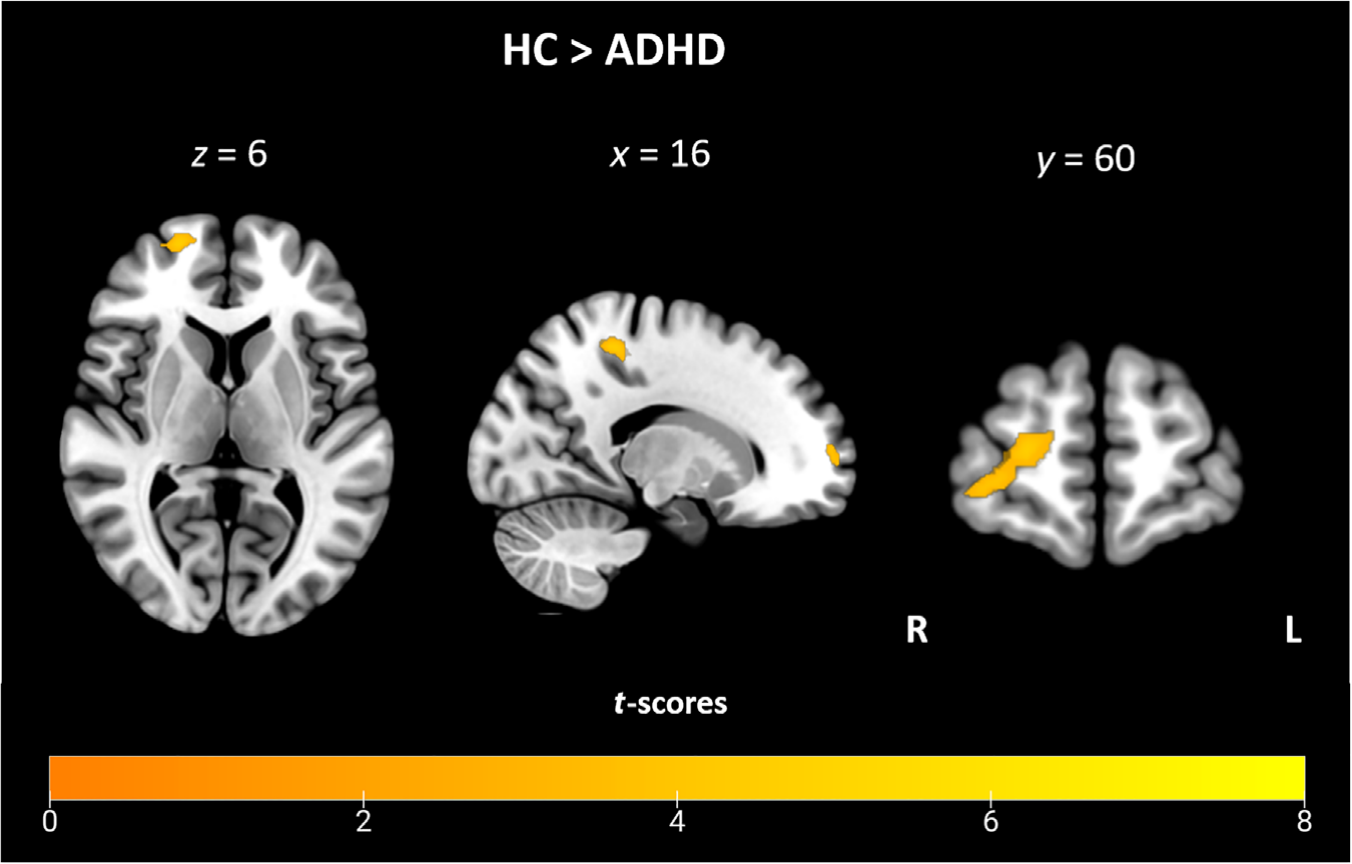
Brain activation associated with anticipation of a decision‐making for the contrast HC > ADHD (*p* < 0.05, FWE‐corrected on cluster level, initial voxel threshold 0.001 uncorrected).

**FIGURE 2:**
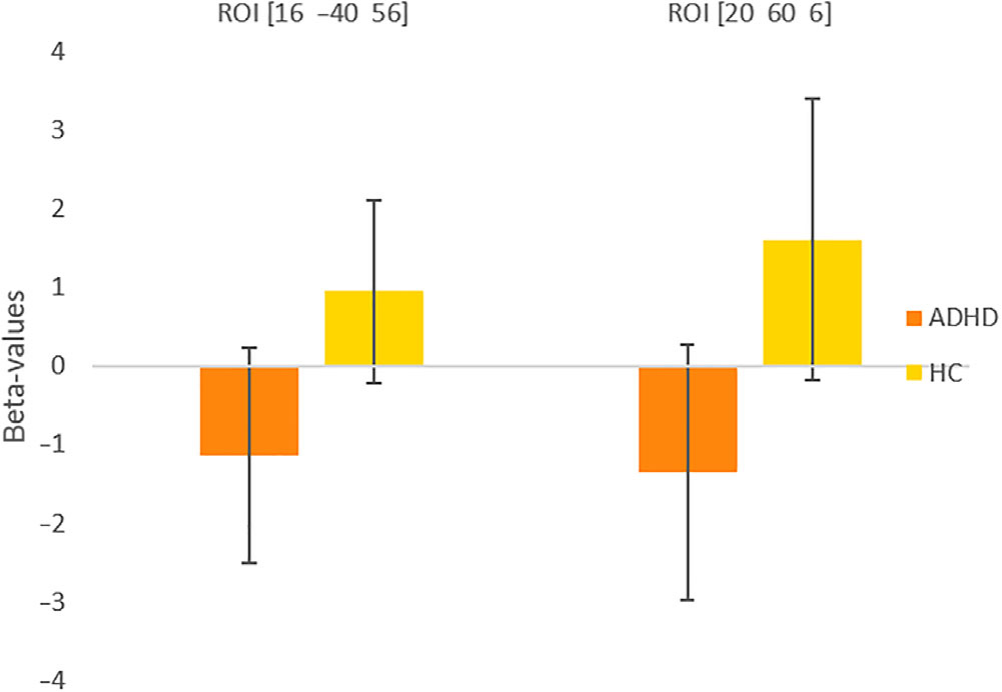
Mean beta values (±standard deviation = error bars) of peak coordinates in the two significant cluster comparing neural activation between ADHD (orange) and HC (yellow).

To identify alterations in the functional activity pattern in the patient group compared to HC, the between‐group contrasts (HC > ADHD and ADHD > HC) for the anticipation phase were analyzed. The whole brain analysis revealed significantly higher activation in HC compared to ADHD, whereas no increased activation patterns were found in ADHD compared to HC. Increased activation in HC compared to ADHD was found in two clusters with peak activation in the right precuneus and the right superior frontal gyrus (see Figure 1; Table 2). Post hoc power analysis using a two‐tailed independent samples *t*‐test with an alpha level of 0.05 revealed a moderate power of Cohen's *d* = 0.56 for peak activation in the right precuneus and 0.69 in the right superior frontal gyrus. Moreover, the cluster with peak activation in the right precuneus extended into the region of the right paracentral lobule (see Table S1). To evaluate whether these group differences were driven by hyperactivation in the HC group or hypoactivation in the ADHD group, peak beta values of both groups were extracted and compared for illustrative purposes (see Figure 2). The results indicate increased activation in the HC group, whereas activation appears to decrease during DM anticipation in the ADHD group. Peak beta values were also found to be negatively correlated with the total score of the CAARS in both the right precuneus (*r* = −0.636, *p* < 0.001) and the right superior frontal gyrus (*r* = −0.611, *p* < 0.001), indicating that greater ADHD symptom severity is associated with reduced neural activation in these regions. Furthermore, no significant correlation was found between TD motion parameters and peak beta values in either the right precuneus (*r* = −0.191, *p* = 0.252) or the right superior frontal gyrus (*r* = −0.257, *p* = 0.119), indicating that motion did not influence the observed group differences in neural activation.

**FIGURE 3:**
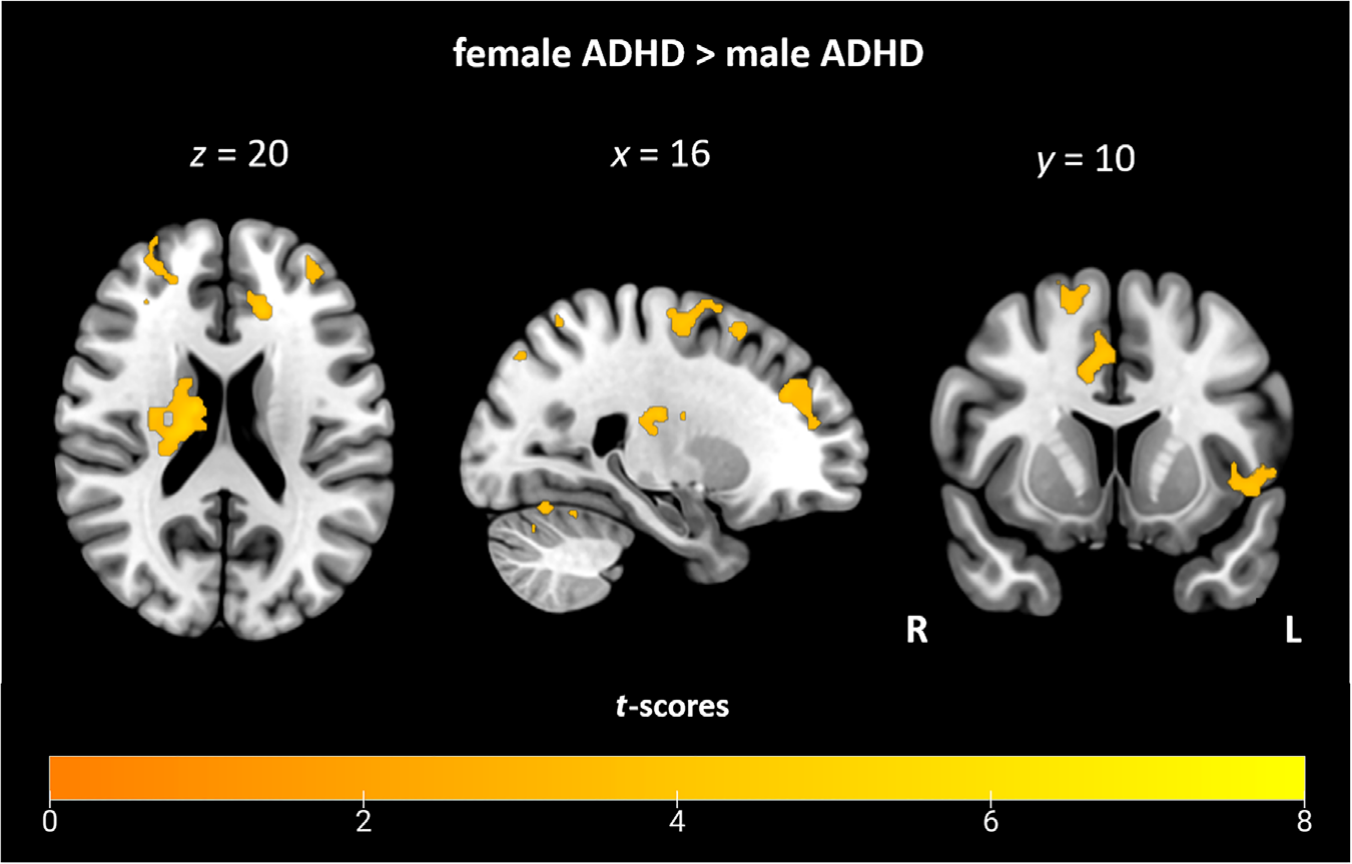
Brain activation associated with anticipation of a decision‐making in the contrast female > male of the within group comparison of the full factorial analysis in ADHD (*p* < 0.05, FWE‐corrected on cluster level, initial voxel threshold 0.001 uncorrected).

An additional exploratory analysis was performed to identify the effects of sex during the anticipation of a DM. Therefore, a whole‐brain full factorial analysis was performed with group and sex as between‐subject factors. The analysis revealed no significant interaction effect between group and sex, indicating that the differences in neuronal activity between groups were not significantly affected by sex. However, according to our previous study results, it was of particular interest to investigate sex differences within the ADHD group, as we hypothesized that sex might play a role in the neural activity of patients with ADHD. For this purpose, we further examined sex differences within the groups (see Table 3). In females compared to males with ADHD, we observed increased neural activity during anticipation of a DM in various clusters located in the frontal lobe, including bilateral dorsolateral and bilateral middle frontal parts (see Figure 3). Further significant activation patterns were found in the left insula, right cuneus, and precuneus, as well as the left precentral and lingual gyrus. Subcortical activation patterns were found in the region of the right caudate (see Table S2). No significant group differences were found when comparing ADHD and HC within females and males separately.

**TABLE 3:** Sex‐related brain activation patterns associated with the anticipation of decision‐making. Displayed are the main effects of sex and within‐group sex comparisons, as revealed by the full factorial analysis with sex and group as between‐subject factors.

	Peak MNI coordinates			*p*_FWE‐corr_ (cluster level)	*Z*_E_ (peak‐level)	Cluster size
	*x*	*y*	*z*			
ADHD_female > male_						
R. Lobule VI of cerebellar hemisphere	8	−78	−18	< 0.001	5.14	335
R. Crus I of cerebellar hemisphere	42	−42	−34	< 0.001	4.87	459
R. Caudate	22	−18	22	< 0.001	4.86	637
R. Superior frontal gyrus, dorsolateral	10	30	28	< 0.001	4.81	1512
L. Insula	−48	2	−4	0.002	4.67	216
R. Cuneus	10	−74	36	< 0.001	4.44	296
L. Superior frontal gyrus, dorsolateral	−16	30	22	0.011	4.41	157
R. Middle frontal gyrus	30	36	28	< 0.001	4.41	484
L. Lobule VIII of cerebellar hemisphere	−36	−44	−46	0.002	4.34	211
Lobule VI of vermis	8	−66	−36	0.001	4.27	226
L. Precentral gyrus	−36	0	62	< 0.001	4.26	455
R. Precuneus	−6	−68	58	< 0.001	4.25	943
L. Lingual gyrus	−18	−64	−14	0.008	4.15	169
L. Middle frontal gyrus	−34	54	−14	< 0.001	4.14	266
L. Supramarginal gyrus	−54	−14	28	0.023	3.97	135
L. Crus I of cerebellar hemisphere	−30	−66	−24	0.002	3.86	212
ADHD_male > female_						
	Not significant					
HC_female > male_						
	Not significant					
HC_male > female_						
	Not significant					

*Note*: Initial voxel threshold was set as uncorrected (*p* < 0.001) for the full factorial analysis. Reported activation patterns are FWE‐corrected (*p* < 0.05) on cluster level. See Supporting Information for further details of significant clusters.

Abbreviations: L, left hemisphere; R, right hemisphere.

## 4 Discussion

The current study is the first to examine whether adult patients with ADHD demonstrate altered neural activity before making a risky decision. Using fMRI during the performance of a modified version of the BART, we found significantly lower activation in the right precuneus and right superior frontal gyrus in adult patients with ADHD compared to HC. Moreover, we explored whether there were sex‐dependent differences during the anticipation phase of a DM. Results indicated no effect of sex in the HC group, whereas in females compared to males with ADHD, neural activity was increased within the DLPFC, left insula, right caudate, right cuneus, and precuneus. These results provide evidence that individuals with ADHD exhibit altered neural activity immediately before making a decision, which may suggest impaired unconscious affectively driven DM behavior.

In the context of the somatic marker hypothesis, specific neural correlates have been reported, with particular emphasis on the vmPFC and OFC, as key regions critical for the integration processes underlying somatic marker functioning (Bechara et al. 2005; Verdejo‐García and Bechara 2009). However, in the current study, we did not observe significant activation differences in these regions between individuals with ADHD and HC. Instead, we identified differences in activation patterns of the right precuneus and the right superior frontal gyrus during the anticipation of a DM. A possible explanation for this discrepancy could be that the majority of studies addressing the somatic marker hypothesis have been based on the IOWA Gambling Task. The paradigm used in the current study was a modified version of the BART, which has not yet been utilized in any neuroimaging studies. In contrast to the original BART, the modified version was designed to favor more intuitive DM behavior (Henn et al. 2023). Since it does not require a choice between two possible options, it likely involves lower cognitive–analytical functioning. On the other hand, in patients with ADHD, we found decreased activation in the right superior frontal gyrus, a region known to be involved in various cognitive functions, including reward anticipation, response inhibition, and cognitive control (Friedman and Robbins 2022; Geier et al. 2010; Ridderinkhof et al. 2004). Furthermore, the superior frontal gyrus has also been identified as a crucial component in the integration of cognitive and affective information (Eshel et al. 2007; Gray, Braver, and Raichle 2002; Ridderinkhof et al. 2004; Van Leijenhorst et al. 2010). However, neural correlates in the preceding processes of DM are largely unexplored in ADHD. Notably, previous research has identified reduced volume and impaired connectivity of the superior frontal gyrus in ADHD (Vilgis et al. 2016; Zhao et al. 2020). Consequently, the results of the current study suggest that impairments in affectively driven DM in ADHD may be more likely associated with hypoactivity in the superior frontal gyrus.

In addition, we observed hypoactivity in the right precuneus in patients with ADHD. The right precuneus has been shown to be involved in functions such as self‐reflection and awareness in previous studies (Farrow et al. 2001; Johnson et al. 2002; Matthys et al. 2012). It has also been implicated in accessing interoceptive states and the experience of emotions (Terasawa, Fukushima, and Umeda 2013). Importantly, the right precuneus is part of the neural network responsible for metacognitive abilities. Specifically, its functional connectivity with the medial anterior PFC has been shown to be related to the metacognitive assessment of memory (Baird et al. 2013; McCurdy et al. 2013). This suggests that the right precuneus plays a crucial role in our awareness of internal sensations, emotional experiences, and metacognitive reflection. In the context of the somatic marker hypothesis, the right precuneus appears to be relevant due to its involvement in the integration of emotion, somatic perception, and memory—all of which are critical components of affectively driven DM. A dysfunctional interaction in these processes can explain disadvantageous DM behavior (Damasio 1996). Furthermore, our findings of decreased activity in the right precuneus align with previous research, indicating reduced gray matter volume in this region among individuals with ADHD. This reduced neural activity may contribute to deficient neurocognitive abilities, potentially leading to behavioral impairments in ADHD (Noordermeer et al. 2017; Noordermeer, Luman, and Oosterlaan 2016).

In summary, both the right precuneus and the right superior frontal gyrus have been shown to be involved in important functions in the context of somatic marker processing. Our findings of decreased neural activity in these regions in individuals with ADHD suggest potential mechanisms underlying impaired affectively driven DM. Moreover, our study highlights the relevance of these regions even before conscious DM, shedding light on subconscious processes that may contribute to DM impairments in ADHD. However, both regions are part of broader networks of brain regions involved in higher‐order cognitive functions, and further research on functional connectivity is needed to reveal further insights into subconscious processes in ADHD.

In our exploratory analysis, we also found that neural activity during the anticipatory phase of DM depends on sex in patients with ADHD. Activation in females was increased compared to males, with the most pronounced differences noted in regions implicated in the neurobiology of ADHD. Specifically, the DLPFC and the insula are frequently considered as neural bases for impairments in executive function as well as emotional regulation (Arnsten and Rubia 2012; Lemiere et al. 2012; Norman et al. 2016; Seidman, Valera, and Makris 2005). Furthermore, sex differences in activation patterns were also observed in the right precuneus, suggesting that the hypoactivity found in the group comparison was primarily driven by males with ADHD. This aligns with existing literature indicating an increased tendency for risk‐taking in males compared to females (Barasinska, Badunenko, and Schäfer 2009; Byrnes, Miller, and Schafer 1999; Eckel and Grossman 2008). However, these sex differences were evident only in the ADHD group and were not identified in the HC group. Consequently, it could be assumed that males with ADHD experience more pronounced neural dysfunction and related ADHD symptoms. This observation contrasts with our initial hypothesis that women, due to increased impairment of affective functions, would exhibit stronger neural dysfunctions during the anticipation phase. However, a study examining the neural relationship between the somatic marker hypothesis and various emotional intelligence parameters showed a negative correlation in this context. Individuals with better abilities in emotional competence exhibited reduced functional activation during performance and less reactivity in response to emotional stimuli, indicating a greater neural efficiency (Killgore and Yurgelun‐Todd 2007). Taken together, the current results suggest sex‐specific differences in neural activity preceding DM, which were only observed in individuals with ADHD. Nevertheless, comparing these findings with existing literature remains challenging as most studies on ADHD still have an unbalanced sex ratio and are predominantly based on male participant data.

For the behavioral parameters of the task, measured by reaction time to DM, we found no significant group differences between HC and ADHD. This indicates that there are no behavioral abnormalities related to risky DM in ADHD. Previous studies have also shown that it is difficult to identify difficulties in real‐life DM in adult patients with ADHD (Dekkers et al. 2016; Mowinckel et al. 2015). In this context, it should be noted that in a meta‐analysis, the effect size for differences between HC and ADHD in gambling tasks was found to be small‐to‐medium (Dekkers et al. 2020). However, in comparison to other typical DM paradigms, like the IOWA Gambling task, the modified version of the BART involves a dynamic and continuous risk manipulation that simulates a better real‐life and real‐time situation. Furthermore, although the BART is restricted to examining primarily financial domains of DM behavior, a recent review has shown that the BART is the most sensitive task for detecting risk‐taking behavior in psychiatric disorders (Harmon, Haas, and Peterkin 2021). Moreover, as demonstrated by our previous studies, we were able to indicate a relation between anticipatory physiological changes, reflecting affective functionality, and subsequent behavior in the modified version of the BART. These findings suggest that affective processes have an impact on the DM using this version of the BART, aligning with the theories and empirical evidence supporting the somatic marker hypothesis (Halbe et al. 2023, 2024). Consequently, performance on the BART may not reveal differences in the objective measurement of behavioral patterns (e.g., reaction time) between individuals with ADHD and HC. However, the underlying processes (e.g., physiological activity) related to behavioral performance may still differ between groups. Taken together, these inhomogeneities highlight the need for further research in the field of impairments in DM behaviors in ADHD. Thus, the findings of the current study indicate that, in particular, unconscious underlying processes and the integration of physiology and emotion may be an essential component for future research.

Considering the current findings, there are some limitations that need to be taken into account. The exploratory full factorial analysis examining the effects of sex on neural activity was based on only a small sample size in the four subgroups due to the two factors included. Possibly, this limited sample size could also have contributed to the lack of a significant effect found in the interaction of sex and group. Consequently, the results should be interpreted cautiously and can only serve as preliminary indicators of a potential effect. It should also be noted that the average age of the participants was in the range of younger adults, which restricts the generalizability of the results to middle‐aged and older patients. Furthermore, it must be considered that ADHD is a heterogeneous disorder with individual variations in symptoms and their impacts, so the current sample may not fully represent the entire population of individuals with ADHD, and further studies with larger sample sizes are needed. Considering the heterogeneity of ADHD, participants have taken various ADHD‐specific medications, some of which may have effects lasting longer than the 24‐h washout period used in this study. Consequently, residual effects from previous medications might not have been entirely eliminated, potentially influencing the study's findings. Future studies should take into account the potential for longer‐lasting medication effects and ensure prolonged washout periods.

Overall, the present study represents the first investigation into neural correlates in patients with ADHD in anticipation of a DM, aiming to provide deeper insights into risky behavior. The current findings suggest that more attention should be given to the right precuneus and superior frontal gyrus concerning metacognitive functioning and interoceptive awareness. Furthermore, the results underscore the complexity of ADHD and the importance of considering sex‐specific neurobiological factors in research and clinical practice. However, further research is needed to explore additional neural processes involved in the interplay between emotion, physiology and DM, as well as to gain further insights into sex‐dependent differences. In this context, future studies should emphasize the examination of integrative processes during the anticipation of behavioral actions. Combining physiological and neuroimaging measures can offer a more comprehensive view of the temporal interaction in these processes.

## Author Contributions

**Eva Halbe**: conceptualization, methodology, formal analysis, visualization, writing–original draft, writing–review and editing, investigation, project administration, data curation. **Alec Jamieson**: software, formal analysis, writing–review and editing, methodology, validation, data curation. **Moritz Bergmann**: investigation, writing–review and editing, methodology, data curation. **Aylin Mehren**: software, formal analysis, writing–review and editing, methodology, validation, data curation. **Ben J. Harrison**: supervision, writing–review and editing, conceptualization, resources, validation. **Christopher G. Davey**: supervision, writing–review and editing, funding acquisition, resources. **Tony St**ö**cker**: software, methodology, writing–review and editing, resources, data curation. **Alexandra Philipsen**: supervision, funding acquisition, writing–review and editing, resources. **Silke Lux**: conceptualization, project administration, writing–review and editing, validation, resources, supervision.

## Ethics Statement

The studies involving human participants were reviewed and approved by the Ethics Committee of the Medical Faculty of the University of Bonn.

## Consent

The patients/participants provided their written informed consent to participate in this study.

## Conflicts of Interest

Alexandra Philipsen declares that she served on advisory boards, gave lectures, performed phase‐3 studies, and received travel grants within the last 5 years from MEDICE Arzneimittel, Pütter GmbH and Co KG, Takeda, Boehringer, Janssen‐Cilag, and receives royalties from books published by Elsevier, Hogrefe, Schattauer, Kohlhammer, Karger, Oxford Press, Thieme, Springer, Schattauer. The other authors declare no conflicts of interest.

### Peer Review

The peer review history for this article is available at https://publons.com/publon/10.1002/brb3.70215

## Supporting information




**Supplementary Figure 1**. Brain activation associated with anticipation of a decision‐making. Within‐group activation patterns for (A) healthy controls (HC) and (B) patients with ADHD (*p* < 0.05, FWE‐corrected on cluster level, initial voxel threshold 0.001 uncorrected).
**Supplementary Table 1** Brain activation during the anticipation of decision‐making for patients with ADHD and healthy controls
**Supplementary Table 2** Gender comparison of brain activation during the anticipation of decision‐making within the patient group

## Data Availability

The data that support the findings of this study are available from the corresponding author upon reasonable request.
