# Supplementary material for: Neural Correlates and Sex‐Specific Effects of Affectively Driven Processes Underlying Decision‐Making in Adult ADHD
Source: Brain Behav. 2025 Feb 28;15(3):e70215. doi: 10.1002/brb3.70215 (PMC11870793; doi:10.1002/brb3.70215)
Supplement: Supplementary file 1 — Supplementary Figure 1. Brain activation associated with anticipation of a decision‐making. Within‐group activation patterns for (A) healthy controls (HC) and (B) patients with ADHD (p < 0.05, FWE‐corrected on cluster level, initial voxel threshold 0.001 uncorrected). Supplementary Table 1 Brain activation during the anticipation of decision‐making for patients with ADHD and healthy controls Supplementary Table 2 Gender comparison of brain activation during the anticipation of decision‐making within the patient group [file BRB3-15-e70215-s001.docx]

Supplementary Material

**Neural correlates and gender-specific effects of affectively driven processes underlying decision-making in adult ADHD.**

Halbe, E., Jamieson, A., Bergmann, M., Mehren, A., Harrison, B.J., Davey, C. G., Stöcker, T., Lux, S, Philipsen, A.


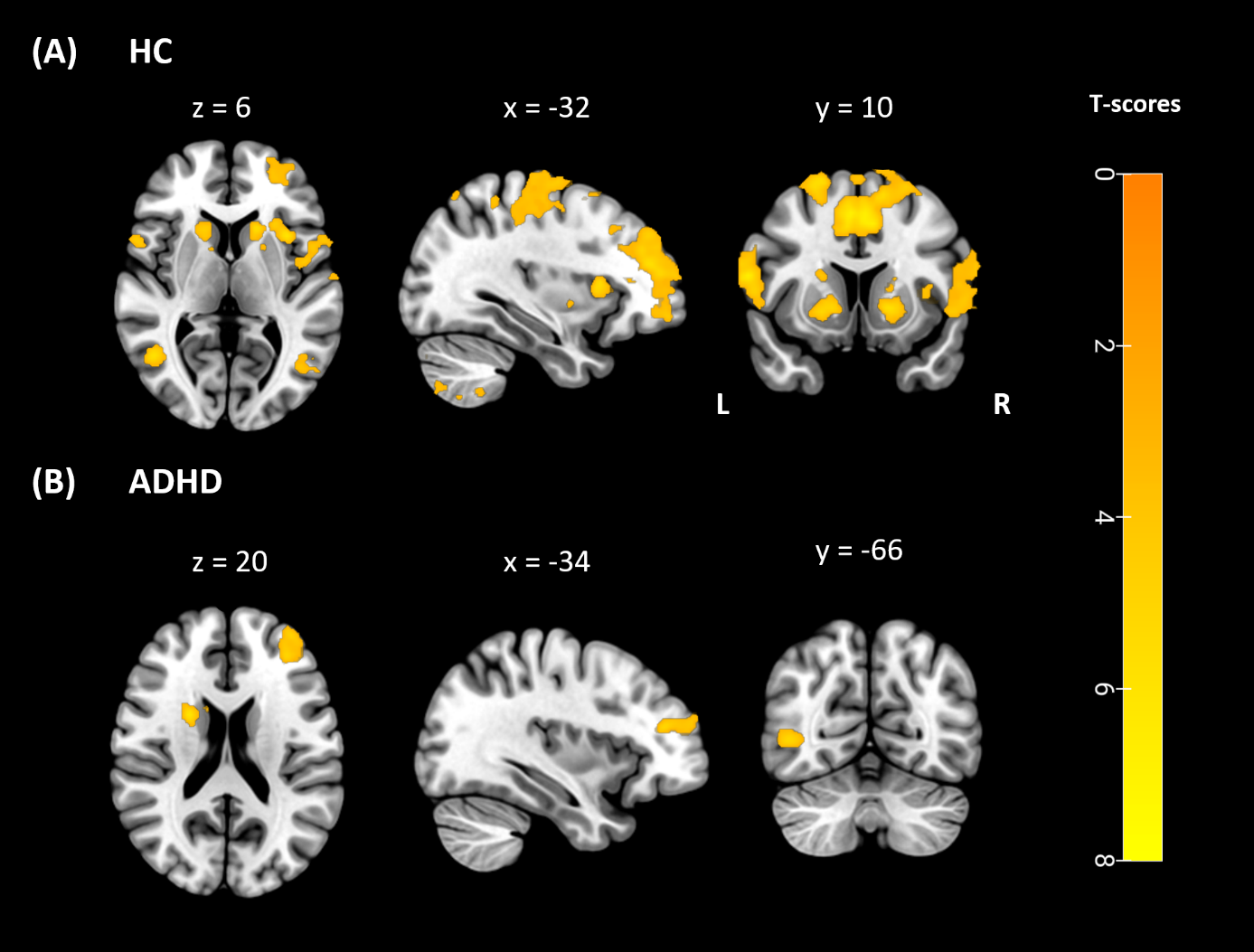


**Supplementary Figure 1.** Brain activation associated with anticipation of a decision-making. Within-group activation patterns for (A) healthy controls (HC) and (B) patients with ADHD (p < 0.05, FWE-corrected on cluster level, initial voxel threshold 0.001 uncorrected).

**Supplementary Table 1**

Brain activation during the anticipation of decision-making for patients with ADHD and healthy controls

| **Contrast** | **Region of peak activation** | **Regions comprised in the cluster** | **MNI coordinates (x, y, z)** | **Cluster size** | **t-statistic** | **z-statistic** | **p*** |
| --- | --- | --- | --- | --- | --- | --- | --- |
| **HC** | R. inferior frontal gyrus, opercular part | R. precentral gyrus  R. rolandic operculum  R. inferior frontal gyrus, triangular part | 60, 12, 16 | 335 | 7.44 | 5.75 | <.001 |
|  |  |  |  |  |  |  |  |
|  | L. middle frontal gyrus | L. superior frontal gyrus 2  L. supplementary motor area  L. inferior parietal gyrus, excluding supramarginal and angular gyri  R. supplementary motor area  L. precentral gyrus  L. postcental gyrus  R. superior frontal gyrus 2  L. supramarginal gyrus  R. middle cingulate & paracingulate gyri | 4, 10, 50 | 8343 | 7.4 | 5.73 | <.001 |
|  |  |  |  |  |  |  |  |
|  | L. precentral gyrus | L. Insula  L. inferior frontal gyrus, opercular part  L. rolandic operculum  L. temporal pole: superior temporal gyrus  L. inferior frontal gyrus, triangular part  L. postcental gyrus  L. superior temporal gyrus  L. IFG pars orbitalis | -36, 14, 8 | 1039 | 6.58 | 5.3 | <.001 |
|  |  |  |  |  |  |  |  |
|  | L. lenticular nucleus, putamen | L. caudate nucleus  L. lenticular nucleus, pallidum  L. Amygdala  L. olfactory cortex  L. Hippocampus | -24, 6, -6 | 496 | 6.15 | 5.05 | <.001 |
|  |  |  |  |  |  |  |  |
|  | R. middle temporal gyrus | R. superior temporal gyrus  R. middle occipital gyrus | 46, -60, 6 | 290 | 6.14 | 5.05 | <.001 |
|  |  |  |  |  |  |  |  |
|  | R. postcental gyrus | R. supramarginal gyrus  R. rolandic operculum | -50, -60, 10 | 250 | 6.01 | 4.97 | <.001 |
|  |  |  |  |  |  |  |  |
|  | L. middle temporal gyrus | L. middle occipital gyrus | -50, -60, 10 | 250 | 5.84 | 4.87 | <.001 |
|  |  |  |  |  |  |  |  |
|  | R. caudate nucleus | R. lenticular nucleus, putamen  R. lenticular nucleus, pallidum  R. Amygdala | 15, 8, -2 | 563 | 5.61 | 4.72 | <.001 |
|  |  |  |  |  |  |  |  |
|  | R. middle frontal gyrus 2 | R. superior frontal gyrus 2  R. inferior frontal gyrus, triangular part | 36, 38, 30 | 757 | 5.47 | 4.62 | <.001 |
|  |  |  |  |  |  |  |  |
|  | L. paracentral lobule | L. middle cingulate & paracingulate gyri  R. middle cingulate & paracingulate gyri  R. paracentral lobule  R. supplementary motor area  L. supplementary motor area  L. Precuneus | -6, -30, 60 | 544 | 5.44 | 4.62 | <.001 |
|  |  |  |  |  |  |  |  |
|  | R. Crus II of cerebellar  hemisphere | R. Crus I of cerebellar  hemisphere  R. Lobule VIII of cerebellar  hemisphere  R. Lobule VIIB of cerebellar  hemisphere  R. Lobule VI of cerebellar  hemisphere | 44, -78, -36 | 754 | 5.32 | 4.54 | <.001 |
|  |  |  |  |  |  |  |  |
|  | L. postcental gyrus | L. superior temporal gyrus  L. supramarginal gyrus  L. Heschl’s gyrus  L. rolandic operculum | -66, -14, 10 | 209 | 5.28 | 4.52 | .002 |
|  |  |  |  |  |  |  |  |
|  | L. Crus I of cerebellar  hemisphere | L. Crus II of cerebellar  hemisphere | -52, -54, -34 | 319 | 5.24 | 4.49 | <.001 |
|  |  |  |  |  |  |  |  |
|  | L. Lobule VIII of cerebellar  hemisphere | L. Crus II of cerebellar  hemisphere  L. Lobule VIIB of cerebellar  hemisphere | -24, -64, -54 | 275 | 4.73 | 4.14 | <.001 |
|  |  |  |  |  |  |  |  |
|  | R. postcental gyrus | R. inferior parietal gyrus, excluding supramarginal and angular gyri  R. superior parietal gyrus  R. supramarginal gyrus | 30, -42, 52 | 123 | 4.63 | 4.07 | .031 |
|  |  |  |  |  |  |  |  |
|  | R. precentral gyrus | R. middle frontal gyrus 2  R. superior frontal gyrus 2 | 38, -14, 60 | 128 | 4.61 | 4.06 | .026 |
|  |  |  |  |  |  |  |  |
| **ADHD** | R. middle cingulate & paracingulate gyri | L. supplementary motor area  R. caudate nucleus  R. supplementary motor area | 22, 6, 20 | 241 | 6.59 | 5.3 | <.001 |
|  |  |  |  |  |  |  |  |
|  | L. middle frontal gyrus 2 | L. superior frontal gyrus 2 | -34, 40, 18 | 231 | 5.57 | 4.7 | <.001 |
|  |  |  |  |  |  |  |  |
|  | R. middle temporal gyrus | R. middle occipital gyrus | 46, -66, 2 | 133 | 5.46 | 4.63 | .021 |
|  |  |  |  |  |  |  |  |
|  |  |  |  |  |  |  |  |
| **HC > ADHD** | R. Precuneus | R. paracentral lobule  R. postcental gyrus  R. superior parietal gyrus | 16, -40, 56 | 154 | 5.05 | 4.63 | .01 |
|  |  |  |  |  |  |  |  |
|  | R. superior frontal gyrus 2 | R. Frontal Sup Medial  R. middle frontal gyrus 2 | 20, 60, 6 | 115 | 4.85 | 4.23 | .041 |
|  |  |  |  |  |  |  |  |

*FWE-corrected on cluster level (initial voxel threshold .001 uncorrected)

**Supplementary Table 2**

Gender comparison of brain activation during the anticipation of decision-making within the patient group

| **Contrast** | **Region of peak activation** | **Regions comprised in the cluster** | **MNI coordinates (x, y, z)** | **Cluster size** | **t-statistic** | **z-statistic** | **p*** |
| --- | --- | --- | --- | --- | --- | --- | --- |
| **ADHD female > ADHD male** | R. Lobule VI of cerebellar  hemisphere | Lobule VI of vermis  Lobule VII of vermis  R. Crus I of cerebellar  hemisphere  R. lingual gyrus  R. Lobule IV, V of cerebellar  hemisphere  L. Crus I of cerebellar  hemisphere  R. fusiform gyrus  L. Crus II of cerebellar  hemisphere | 8,-78,-18 | 335 | 6.38 | 5.14 | <.001 |
|  |  |  |  |  |  |  |  |
|  | R. Crus I of cerebellar  hemisphere | R. Lobule VI of cerebellar  hemisphere  R. inferior temporal gyrus  R. Fusiform  R. Crus II of cerebellar  hemisphere | 42, -42, -34 | 459 | 5.91 | 4.87 | <.001 |
|  |  |  |  |  |  |  |  |
|  | R. caudate nucleus | R. Thalamus, ventral lateral  R. Thalamus, pulvinar medial  R. Thalamus, mediodorsal medial magnocellular  L. Thalamus, pulvinar medial  R. Thalamus, ventral posterolateral  R. Thalamus, lateral posterior  L. Thalamus, mediodorsal medial magnocellular  R. Thalamus, pulvinar anterior  L. Thalamus, pulvinar anterior  R. Thalamus, anteroventral nucleus  R. Thalamus, pulvinar lateral  R. Thalamus, mediodorsal lateral parvocellular  L. Thalamus, mediodorsal lateral parvocellular  L. Thalamus, ventralposterolateral  L. Thalamus, lateral posterior  L. Hippocampus | 22, -18, 22 | 637 | 5.9 | 4.86 | <.001 |
|  |  |  |  |  |  |  |  |
|  | R. Frontal Sup 2 | R. supplementary motor area  R. middle cingulate & paracingulate gyri  L. supplementary motor area  L. middle cingulate & paracingulate gyri  R. Anterior cingulate cortex,  supracallosal  R. middle frontal gyrus 2  R. precentral gyrus  L. superior frontal gyrus, medial  L. anterior cingulate cortex,  supracallosal | 10, 30, 28 | 1512 | 5.81 | 4.81 | <.001 |
|  |  |  |  |  |  |  |  |
|  | L. Insula | L. temporal pole: superior temporal gyrus  L. superior temporal gyrus  L. rolandic operculum  L. inferior frontal gyrus, opercular part  L. inferior frontal gyrus, triangular part  L. IFG pars orbitalis | -48, 2, -4 | 216 | 5.58 | 4.67 | .002 |
|  |  |  |  |  |  |  |  |
|  | R. Cuneus | R. Precuneus  R. superior occipital gyrus  R. superior parietal gyrus | 10, -74, 36 | 296 | 5.22 | 4.44 | <.001 |
|  |  |  |  |  |  |  |  |
|  | L. superior frontal gyrus 2 | L. superior frontal gyrus, medial  R. inferior frontal gyrus, opercular part | -16, 30, 22 | 157 | 5.17 | 4.41 | .011 |
|  |  |  |  |  |  |  |  |
|  | R. middle frontal gyrus 2 | R. superior frontal gyrus 2  R. inferior frontal gyrus, opercular part | 30, 36, 28 | 484 | 5.17 | 4.41 | <.001 |
|  |  |  |  |  |  |  |  |
|  | L. Lobule VIII of cerebellar  hemisphere | L. Lobule VIIB of cerebellar  hemisphere  L. Lobule VI of cerebellar  hemisphere  L. Crus I of cerebellar  hemisphere  L. Crus II of cerebellar  hemisphere | -36, -44, -46 | 211 | 5.06 | 4.34 | .002 |
|  |  |  |  |  |  |  |  |
|  | Lobule VI of vermis | R. Lobule VIII of cerebellar  hemisphere  Lobule VIII of vermis  Lobule IX of vermis  R. Lobule IX of cerebellar  hemisphere  Lobule IV, V of vermis  R. Lobule VI of cerebellar  hemisphere  L. Lobule IV, V of cerebellar  hemisphere  R. Crus I of cerebellar  hemisphere  Lobule X of vermis | 8, -66, -36 | 226 | 4.96 | 4.27 | .001 |
|  |  |  |  |  |  |  |  |
|  | L. precentral gyrus | L. postcental gyrus  L. superior frontal gyrus 2  L. superior frontal gyrus  L. middle frontal gyrus 2  L. Paracentral Lobule | -36, 0, 62 | 455 | 4.95 | 4.26 | <.001 |
|  |  |  |  |  |  |  |  |
|  | R. Precuneus | L. Precuneus  L. middle cingulate & paracingulate gyri  R. superior parietal gyrus  R. middle cingulate & paracingulate gyri  L. Paracentral Lobule  R. Paracentral Lobule  R. posterior cingulate gyrus | -6, -68, 58 | 943 | 4.93 | 4.25 | .002 |
|  |  |  |  |  |  |  |  |
|  | L. Lingual | L. Lobule VI of cerebellar  hemisphere | -18, -64, -14 | 169 | 4.78 | 4.15 | .008 |
|  |  |  |  |  |  |  |  |
|  | L. middle frontal gyrus 2 | L. inferior frontal gyrus, triangular part  L. superior frontal gyrus 2 | -34, 54, 14 | 266 | 4.77 | 4.14 | <.001 |
|  |  |  |  |  |  |  |  |
|  | L. supramarginal gyrus | L. postcental gyrus  L. superior temporal gyrus  L. inferior parietal gyrus, excluding supramarginal and angular gyri  L. rolandic operculum | -54, -14, 28 | 135 | 4.52 | 3.97 | .023 |
|  |  |  |  |  |  |  |  |
|  | L. Crus I of cerebellar  hemisphere | L. Lobule VI of cerebellar  hemisphere  L. interior temporal gyrus  L. Fusiform | -30, -66, -24 | 212 | 4.36 | 3.86 | .002 |
|  |  |  |  |  |  |  |  |

*FWE-corrected on cluster level (initial voxel threshold .001 uncorrected)
